# Biological variation in the serum and urine kidney injury markers of a healthy population measured within 24 hours

**DOI:** 10.1186/s12882-022-02819-2

**Published:** 2022-05-24

**Authors:** Li-Rui Kong, Fei Wei, Da-Hai He, Chao-Qiong Zhou, Hong-chuan Li, Feng Wu, Yu Luo, Jian-wei Luo, Qian-rong Xie, Hai Peng, Yan Zhang

**Affiliations:** 1 Traditional Chinese Medicine Hospital of Pidu District, No. 342, South Street, Pidu District, Chengdu, Sichuan 611730 People’s Republic of China; 2 Clinical Laboratory of Qinghai Provincial People’s Hospital Xining, Xining, Qinghai 810006 People’s Republic of China

**Keywords:** Kidney injury markers, Biological variation, Reference change value, Individual index

## Abstract

**Background and aims:**

To explore the biological variation (BV) of kidney injury markers in serum and urine of healthy subjects within 24 hours to assist with interpretation of future studies using these biomarkers in the context of known BV.

**Materials and methods:**

Serum and urine samples were collected every 4 hours (0, 4, 8, 12, 16 and 20 hours) from 31 healthy subjects within 24 hours and serum creatinine (s-Crea), serum β2-microglobin (s-β2MG), serum cystatin C (s-CYSC), serum neutrophil gelatinase-associated lipoprotein (s-NGAL), urine creatinine (u-Crea), urine β2-microglobin (u-β2MG), urine cystatin C (u-CYSC), urine neutrophil gelatinase-associated lipoprotein (u-NGAL) were measured. Outlier and variance homogeneity analyses were performed, followed by CV-ANOVA analysis on trend-corrected data (if relevant), and analytical (CV_A_), within-subject (CV_I_), and between-subject (CV_G_) biological variation were calculated.

**Results:**

The concentration of kidney injury markers in male was higher than that in female, except for u-CYSC and u-NGAL. There were no significant difference in serum and urine kidney injury markers concentration at different time points. Serum CV_I_ was lower than urine CV_I_, serum CV_G_ was higher than CV_I_, and urine CV_G_ was lower than CV_I_. The individual index (II) of serum kidney injury markers was less than 0.6, while the II of urinary kidney injury markers was more than 1.0.

**Conclusions:**

This study provides new short-term BV data for kidney injury markers in healthy subjects within 24 hours, which are of great significance in explaining other AKI / CKD studies.

## Introduction

The causes of kidney injury are diverse, and the underlying mechanisms are complex. According to the cause of the disease, kidney injury can be divided into acute kidney injury (AKI) and chronic kidney disease (CKD). The global incidence of AKI in hospitalized patients ranges from 3.0% to 18.3 % [1], however the incidence in hospitalized patients in China ranges from 6.9 % to 11.6 % [2, 3]. Currently, the clinical diagnosis of AKI is based on the standards set by “Kidney Disease: Improving Global Outcomes” (KDIGO) [4]. Acute kidney injury is defined as follows: (1) an increase in serum creatinine (s-Crea) by 26.5 μmol/L (0.3 mg/dL) within 48 hours; or (2) an increase in s-Crea that is 1.5~2 times the baseline value within 7 days; or (3) urine output of < 0.5 mL kg^–1^ h^–1^ for more than 6 hours. Although the diagnostic window is advanced to 48 hours, changes in s-Crea levels are affected by body weight, age, sex, drugs, muscle content, and protein intake; furthermore, when the glomerular filtration rate (GFR) is significantly reduced to below 50%, the s-Crea level begins to increase. In addition, urine output is easily affected by physiological reactions, pathological factors, and drugs, such as diuretics; therefore, the sensitivity and specificity of the KDIGO standard in diagnosing AKI are not high, and it is particularly important to find biomarkers for the early diagnosis of AKI.

A new marker, β_2_-microglobulin (β_2_MG), can be used as a potential serum marker for a GFR. An increase in urinary β_2_MG (u-β_2_MG) excretion can be used as an indicator of kidney tubular injury, and this might have new value in evaluating glomerular filtration function and kidney tubular diseases. Cystatin C (CYSC) is a good marker that reflects glomerular dysfunction faster and is more sensitive than Crea [5]. CYSC can be freely filtered by the kidney, reabsorbed, and degraded in the proximal convoluted tubule of the glomerulus. Moreover, CYSC is not excreted by the kidney tubule. CYSC can meet the requirements of ideal endogenous GFR markers and is a new sensitive index for evaluating GFR [6]. Furthermore, human neutrophil gelatinase-associated lipocalin (NGAL) is helpful in identifying early kidney tubular injuries. The expression of NGAL in serum and urine increases significantly after 2 hours, and this occurs earlier than that of other markers; thus, NGAL is considered one of the most effective early markers of AKI [7, 8]. However, differences in the diagnosis of AKI are due to the progression of the disease and/or the change in the treatment, rather than nonspecific variations among individuals. Therefore, it is important to study the biological variation (BV) of kidney injury markers in a healthy population within 24 hours.

The European Federation of Clinical Chemistry and Laboratory Medicine (EFLM) conducts a meta-analysis of the biological variation estimates of biomarkers, and establishes various biomarker biological variation databases for interpreting clinical experimental results and defining analytical performance specifications [9–11]. Currently, research on kidney markers locally and in other counties mainly focuses on the diurnal biological variation in the disease state [12–14] . There have not been reports on the standardized biological variation in serum and urine samples of a healthy population within 24 hours. Jonker et al. published a research review on kidney marker BV [15]. Only some kidney markers obtained valuable BV data, such as CYSC, and further research is needed to obtain more data. Thus, the purpose of this study was to determine the distribution levels of Crea, β_2_MG, CYSC, and NGAL in the serum and urine of healthy individuals within 24 hours, and to study the biological variation in the biomarkers from the collected samples.

## Material and methods

### Study population and protocols

Samples were collected from 31 healthy volunteers from September to November 2019 in the Chengdu Pidu Traditional Chinese Medicine Hospital, including 17 males (median age, 30 years; range, 21–54 years) and 14 females (median age, 33 years; range, 18–48 years). There were no significant differences in their age, blood pressure, or heart rate (*P* > 0.05). The inclusion criteria were: healthy individuals without chronic diabetes, hypertension, goiter, cardiovascular and cerebrovascular diseases, no history of medication, and a stable lifestyle. All participants participated in the completion of the questionnaire survey to verify their health status, collect information about lifestyle, and conduct related physical examinations to ensure that all inclusion criteria were met [16]. Participants were fed a normal diet (8:00, 12:00, and 19:00) and had sleep as usual. The research plan was approved by the Ethics Committee of Chengdu Pidu District Hospital of Traditional Chinese Medicine, and written informed consent was obtained from each participant.

### Sample collection and handling

After a time interval of 4 hours (0, 4, 8, 12, 16, and 20 hours), six serum samples were collected within 24 hours. The serum had naturally precipitated from the whole blood samples after being placed at room temperature for 30-90 min. The blood was centrifuged at 3,000 *g* for 10 min, and the serum was transferred to an Eppendorf tube and was stored at -70 °C until it was analyzed. Urine was collected at the same time point, packed in Eppendorf tube, and stored in a -70 °C freezer for testing.

The specimens were thawed at room temperature, and the concentrations of the bio- markers were determined by the same operator using a Hitachi 7180 automatic biochemical analyzer system. Before testing, the analyzer was calibrated in accordance with the manufacturer's instructions, and all samples of the participants were tested twice. β2MG, CYSC, and NGAL were determined by the latex immunoturbidimetry. Crea was determined by the highly specific sarcosine oxidase method. The measured value of the method can be traced to the highest standard ID-MS method for the determination of Crea, and the measurement performance can meet clinical requirements. Internal quality control uses two levels of quality control products. The batch number of Crea was 48811/45813, which was provided by the Bio-rad Company of the United States. The batch number of β_2_MG and CYSC was 68912, and the batch number of NGAL was 0619021, which were provided by Sichuan Maccura Industrial Company.

### Statistical analysis

Student’s t-test was used to test the difference in average values between male and female groups, and *P* <0.05 was considered to indicate statistical significance. The Shapiro-Wilk test was used to verify whether the test data were normally distributed [17]. Logarithmic transformation of the data with skewed distribution was performed, and the Dixon WJ criterion was used to process outliers [18]. The analytical variation (CV_A_) was obtained from repeated measurement data in the same analysis batch. The Bartlett and Cochran tests [19, 20] were used to calculate within-subject biological variation (CV_I_) and between-subject variation (CV_G_). The 95% confidence interval (CI) was calculated according to Roraas [21]. The reference change value (RCV) and individual index (II) were calculated according to Harris [22]. CV_T_ is the total number within subject’s variation, and Z is the probability of statistical significance (at 95% CI, Z = 1.96), and calculations were performed as follows:

1 $${\mathrm{CV}}_{\mathrm{A}}=\left(\mathrm{SD}\left/ \mathrm{X}\right.\right)\times 100\mathrm{\%}$$

2 $${\mathrm{CV}}_{\mathrm{I}}={\left(\mathrm{C}{{\mathrm{V}}_{\mathrm{T}}}^{2}-\mathrm{C}{{\mathrm{V}}_{\mathrm{A}}}^{2}\right)}^{1\left/ 2\right.}$$

3 $${\mathrm{CV}}_{\mathrm{G}}={\left(\mathrm{C}{{\mathrm{V}}_{\mathrm{T}}}^{2}-\mathrm{C}{{\mathrm{V}}_{\mathrm{I}}}^{2}-\mathrm{C}{{\mathrm{V}}_{\mathrm{A}}}^{2}\right)}^{1\left/ 2\right.}$$

4 $$\mathrm{RCV}={2}^{1\left/ 2\right.}\times \mathrm{z}{(\mathrm{C}{{\mathrm{V}}_{\mathrm{A}}}^{2}+\mathrm{C}{{\mathrm{V}}_{\mathrm{I}}}^{2})}^{1\left/ 2\right.}$$

5 $$\mathrm{II}= \frac{{\mathrm{CV}}_{\mathrm{I}}}{{\mathrm{CV}}_{\mathrm{G}}}$$

## Results

### Minimum–maximum concentration ranges for kidney injury markers

**Fig. 1:**
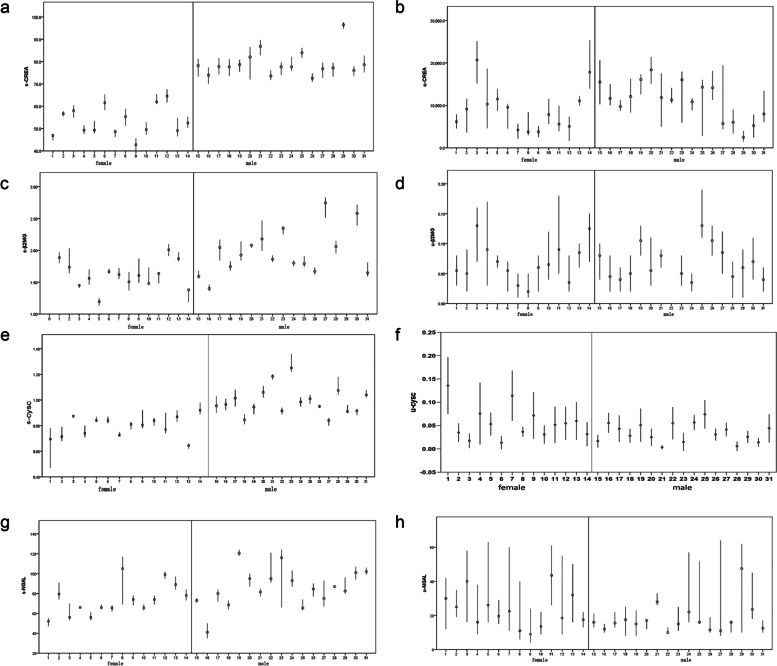
Serum and urine kidney injury markers in females and males. **a**-**b** S-Crea and u-Crea levels in 14 females and 17 males. **c**-**d** S-β2MG and u-β2MG levels in 14 females and 16 males. **e**-**f** S-CYSC and u-CYSC levels in 14 females and 17 males. **g**-**h**) S-NGAL and u-NGAL levels in 14 females and 17 males. The horizontal ordinate title was each individual. The ordinate title was concentration of marker

We collected six samples from 31 people within 24 hours, including 186 serum samples and 186 urine samples. After repeated measurement, 744 results could be analyzed. Within 24 hours, the concentrations of s-Crea, s-β_2_MG, s-CYSC, s-NGAL, u-Crea, u-β_2_MG, u-CYSC, and u-NGAL were skewed. As described in the methods section, outlier detection was performed for each analyte, with No. 29 s-β_2_MG and 22 u-β_2_MG excluded. The changes in the concentrations of biomarkers are shown in Fig. 1.

### Demographic characteristics and concentrations of kidney injury markers of healthy subjects

The characteristics of subjects were presented in Table 1. Baseline characteristics of 31 healthy subjects were not significant, except for BMI. There were significantly difference in serum and urinary kidney injury markers between male and female (*P*<0.05). The serum and urinary kidney injury markers were higher in male than in female, except u-CYSC and u-NGAL. These data are shown in Table 1.

**Table 1:** Characteristics and concentration levels of the kidney injury markers in male and female group

	Male(*N*=17) mean (SD)	Female(*N*=14) mean (SD)	*P*-value
Age, year	33.94(11.44)	31.07(9.83)	0.465
BMI, kg/m^2^	24.01(2.08)	21.31(2.80)	<0.01
Systolic pressure , mmHg	117(12)	108(11)	0.074
Diastolic pressure, mmHg	75(7)	73(7)	0.690
Heart rate, bpm	74(9)	68(9)	0.104
S-Crea, umol/L	83.74 (9.70)	51.40 (9.95)	<0.001
S-β2MG, ug/L	2.03 (0.48)	1.59 (0.32)	<0.001
S-CYSC, mg/L	0.99 (0.13)	0.78 (0.13)	<0.001
S-NGAL, mol/L	97.00 (44.55)	74.10 (24.02)	<0.001
u-Crea, umol/L	12133.41 (9331.81)	9301.20 (6516.58)	<0.05
u-CYSC, mg/L	0.03 (0.03)	0.06 (0.05)	<0.01
u-β2MG, ug/L	0.14 (0.20)	0.07 (0.05)	<0.01
u-NGAL, mol/L	27.88 (26.53)	38.10 (36.12)	<0.05

### Concentrations of kidney injury markers at different collection time points

The levels of s-Crea, s-β_2_MG, s-CYSC, s-NGAL, u-Crea, u-β_2_MG, u-CYSC, and u-NGAL at different collection time points (0, 4, 8, 12, 16 and 20 hours) within 24 hours were not statistically significant (*P* > 0.05). These data are shown in Table 2.

**Table 2:** Concentration levels of the kidney injury markers in serum and urine samples at different time points at 95% confidence interval

Items	Groups	0:00	4:00	8:00	12:00	16:00	20:00	*P*-value
S-CREA, umol/L	All	68.00(61.66-74.34)	68.73(62.92-74.53)	69.13(62.95-75.31)	67.35(58.88-75.83)	71.9(65.14-78.67)	69.69(63.30-76.09)	0.961
	Male	81.94(77.15-86.74)	81.79(77.88-85.71)	82.82(78.88-86.76)	84.82(78.41-91.24)	87.05(82.82-91.29)	84.00(79.94-88.06)	0.774
	Female	51.07(46.98-55.16)	52.86(49.18-56.53)	52.50(47.69-57.31)	46.14(38.20-54.09)	53.50(48.54-58.46)	52.32(47.66-56.98)	0.624
S-β2MG, ug /L	All	1.82(1.65-1.99)	1.97(1.81-2.12)	1.87(1.72-2.02)	1.77(1.57-1.97)	1.76(1.61-1.91)	1.82(1.66-1.97)	0.391
	Male	2.01(1.75-2.27)	2.13(1.90-2.36)	2.04(1.81-2.27)	2.04(1.80-2.29)	1.94(1.72-2.17)	2.02(1.82-2.23)	0.770
	Female	1.59(1.44-1.73)	1.76(1.63-1.90)	1.66(1.54-1.78)	1.44(1.20-1.68)	1.54(1.41-1.68)	1.56(1.39-1.73)	0.160
S-CYSC, mg/L	All	0.90(0.85-0.95)	0.93(0.89-0.98)	0.89(0.84-0.94)	0.85(0.78-0.93)	0.90(0.84-0.95)	0.92(0.87-0.98)	0.694
	Male	0.98(0.91-1.05)	1.00(0.94-1.05)	0.98(0.93-1.03)	0.97(0.92-1.03)	1.00(0.94-1.06)	1.03(0.97-1.09)	0.744
	Female	0.80(0.76-0.85)	0.85(0.81-0.90)	0.78(0.71-0.86)	0.71(0.59-0.83)	0.77(0.72-0.82)	0.79(0.75-0.83)	0.157
S-NGAL, mol/L	All	82.69(67.81-97.58)	84.50(71.83-97.17)	92.77(79.43-106.12)	85.26(74.13-96.38)	84.06(73.98-94.15)	90.63(72.48-108.78)	0.621
	Male	93.29(68.04-118.55)	94.59(73.47-115.70)	96.44(79.10-113.78)	96.21(81.44-110.97)	93.62(77.55-109.68)	107.82(77.25-138.40)	0.803
	Female	69.82(60.36-79.28)	72.25(63.69-80.81)	88.32(67.12-109.53)	71.96(57.42-86.50)	72.46(64.62-80.31)	69.75(62.59-76.91)	0.618
u-CREA, umol/L	All	9430.92(7587.99-11273.85)	9425.18(7042.76-11807.60)	13032.95(11101.06-14964.84)	14084.71(8782.35-19387.06)	8998.60(7131.55-10865.64)	10153.74(7908.49-12398.99)	0.082
	Male	9966.59(7364.86-12568.32)	11798.23(8620.03-14976.44)	13208.53(11132.61-15284.45)	17793.56(9253.33-26333.79)	8570.53(6087.73-11053.33)	11463.03(8048.62-14877.44)	0.141
	Female	8780.46(6132.66-11428.27)	6543.61(3472.29-9614.93)	12819.75(9269.24-16370.26)	9581.11(4709.48-14452.73)	9518.39(6610.78-12426.01)	8563.89(5922.95-11204.83)	0.112
u-β2MG, ug /L	All	0.10(0.05-0.14)	0.11(0.05-0.16)	0.12(0.07-0.18)	0.12(0.05-0.19)	0.10(0.04-0.17)	0.10(0.06-0.13)	0.650
	Male	0.12(0.04-0.20)	0.14(0.04-0.24)	0.15(0.05-0.25)	0.18(0.07-0.30)	0.13(0.01-0.25)	0.12(0.06-0.18)	0.700
	Female	0.07(0.05-0.09)	0.07(0.05-0.09)	0.09(0.06-0.13)	0.05(0.03-0.07)	0.07(0.04-0.10)	0.07(0.06-0.089)	0.281
u-CYSC, mg/L	All	0.04(0.03-0.05)	0.04(0.03-0.06)	0.06(0.04-0.07)	0.04(0.03-0.05)	0.04(0.02-0.05)	0.04(0.03-0.056)	0.493
	Male	0.03(0.02-0.04)	0.04(0.02-0.05)	0.04(0.03-0.05)	0.04(0.02-0.05)	0.02(0.01-0.04)	0.03(0.02-0.05)	0.601
	Female	0.05(0.04-0.07)	0.05(0.02-0.08)	0.08(0.05-0.11)	0.04(0.02-0.07)	0.05(0.03-0.08)	0.06(0.04-0.08)	0.331
u-NGAL, mol/L	All	26.71(18.03-35.39)	38.02(25.13-50.90)	31.42(20.28-42.56)	30.32(20.99-39.66)	32.18(22.13-42.23)	36.32(22.31-50.34)	0.887
	Male	29.35(16.70-42.00)	19.53(13.39-25.67)	19.62(11.61-27.62)	28.23(16.40-40.07)	34.38(17.30-51.46)	36.15(20.33-51.96)	0.395
	Female	23.50(11.65-35.35)	60.46(37.66-83.27)	45.75(25.08-66.42)	32.86(17.60-48.11)	29.50(20.78-38.22)	36.54(11.43-61.64)	0.080

### Components of biological variation, RCVs and IIs for kidney injury markers

The CV_A_, CV_I_, CV_G_, RCV, and II at a 95 % CI for the serum and urine biomarkers of kidney injury showed different individual differences between males and females. These data are shown in Table 3.

**Table 3:** CV_A_, CV_I_, CV_G_, RCV, and II of kidney injury markers within 24 h at 95 % confidence interval

Items	Group	CV_A_%(95%CI)	CV_I_ %(95%CI)	CV_G_%(95%CI)	RCV	II
s-Crea	All	2.72 (2.53-2.93)	7.41 (6.67-8.33)	24.82 (19.83-33.17)	21.88	0.3
	Male		6.91 (6.02-8.11)	7.93 (5.91-12.07)		
	Female		7.84 (6.73-9.40)	14.05 (10.18-22.63)		
s-β2MG	All	2.52(2.34-2.72)	10.27(9.22-11.60)	17.41(13.86-23.40)	29.31	0.59
	Male		9.99 (8.63-11.86)	16.22 (11.98-25.11)		
	Female		10.62 (9.10-12.74)	13.07 (9.47-21.05)		
s-CYSC	All	2.91 (2.71-3.13)	4.70 (4.23-5.29)	14.07 (11.25-18.81)	15.32	0.33
	Male		3.07 (2.68-3.61)	9.65 (7.18-14.68)		
	Female		5.31 (4.54-6.38)	9.06 (6.57-14.60)		
s-NGAL	All	2.65 (2.47-2.86)	11.64 (10.44-13.16)	22.20 (17.74-29.68)	33.09	0.52
	Male		12.09 (10.44-14.35)	21.33 (15.88-32.46)		
	Female		10.77 (9.21-12.96)	19.67 (14.26-31.69)		
u-Crea	All	3.41(3.18-3.68)	47.40(42.64-53.38)	37.58(30.03-50.23)	131.73	1.26
	Male		45.23 (39.30-53.27)	29.96 (22.31-45.59)		
	Female		50.50 (43.30-60.59)	46.45 (33.67-74.83)		
u-β2MG	All	29.41(27.34-31.82)	53.00 (47.46-60.03)	32.17 (25.62-43.24)	168.01	1.65
	Male		52.02 (44.57-62.52)	25.68 (18.97-39.74)		
	Female		53.31 (45.75-63.87)	34.01 (24.65-54.79)		
u-CYSC	All	44.42(41.40-47.93)	59.85 (53.86-67.34)	57.80 (46.19-77.26)	206.59	1.04
	Male		44.25 (38.54-51.96)	52.13 (38.82-79.34)		
	Female		64.09 (54.90-77.02)	52.94 (38.38-85.29)		
u-NGAL	All	8.33 (7.73-9.02)	61.82 (55.22-70.22)	24.46 (19.54-32.69)	172.90	2.53
	Male		64.37 (55.51-76.61)	21.72 (16.18-33.05)		
	Female		58.93 (49.95-71.88)	22.56 (16.36-36.35)		

## Discussion

Biological variation research requires strict time and resource management, and is a challenging study. Our study is the first time to evaluate the serum and urine specimens of kidney injury markers simultaneously within 24 hours. Baseline characteristics of 31 healthy subjects were not significant, except for BMI. There were significant differences in serum and urine kidney injury markers between females and males. Consistent with previous studies, Crea was higher in male than in female [23], while NGAL in urine was higher in female than in male [24, 25]. Contrary to previous studies, our u-CYSC was higher in female than in male [25], while s-β_2_MG was higher in male than in female [26]. The concentration levels of kidney injury markers collected at different time points were not statistical significant. The CV_I_ of serum samples of 31 healthy subjects was around 5–10%, the CV_I_ of urine specimens was above 40%, and the CV_I_ of kidney injury serum markers was lower than urine CV_I_, consistent with the report of related studies [27–31]. Serum CV_G_ was all higher than CV_I_, and urine CV_G_ was lower than CV_I_. CV_I_ is important for personalized reference intervals, and CV_G_ is important for population-based reference intervals. RCV and II calculated using biological variation data influence the application of reference intervals in the clinic.

S-Crea [32–34] is still the basis of KDIGO’s definition and staging of kidney injury. The level of s-Crea is relatively stable, it is not reabsorbed by the kidney tubules, the excreted amount is small, and the measurement is cheap, and thus, it has always been the most extensive clinical test index for evaluating kidney function, but it also has many limitations, including kidney tubular secretion and its related with the muscle mass, age, and sex of patients. Crea is a late-stage and nonspecific marker of kidney injury [35]. In this study, the CV_I_ and CV_G_ of s-Crea within 24 hours were 7.41% (95% CI, 6.67–8.33) and 24.82 % (95% CI, 19.83–33.17), respectively, which were higher than the short-term biological variation reported by Carobene and Judith [36, 37]. The values of CV_I_ and CV_G_ of u-Crea at 24 hours were 47.40% (95% CI, 42.64–53.38) and 37.58% (95% CI, 30.03–50.23), which were higher than the values published in the biological variation database data in 2014 [38], EuBIVAS of the EFLM [11]. The cause of elevated BV was different from the time of specimen collection, and we collected specimens at different time points within 24 hours.

The biomarker β_2_MG is produced, synthesized, and released constantly in individuals with a normal kidney function, and it is then reabsorbed and decomposed through glomerular filtration and in proximal kidney tubules (99%). Although the β_2_MG content in the urine of normal people is very small, β_2_MG is related to many disease processes. s-β_2_MG and u-β_2_MG concentrations are increased in many disease conditions; thus, this might limit the usefulness of β_2_MG as a biomarker for diagnosis. The levels of s-β_2_MG and u-β_2_MG *in vivo* are reliable and easy to detect. Several studies have analyzed the ability of β_2_MG to be used for the evaluation of kidney function and have reported that β_2_MG is superior to s-Crea in detecting changes in kidney function [39]. β_2_MG has been used not only to evaluate kidney tubular function, but also to monitor glomerular function. In this study, the CV_I_ and CV_G_ of s-β_2_MG and u-β_2_MG were 10.27% (95% CI, 9.22–11.60), 17.41 (95% CI, 13.86–23.40), 53.00% (95% CI, 47.46–60.03) and 32.17% (95% CI, 25.62–43.24), respectively. The CV_I_ of s-β_2_MG was higher than the online number reported in 2014 [38], and there was a consistency in the CV_G_. The BV of u-β_2_MG has not been reported.

CYSC is a cysteine protease inhibitor protein produced by nucleated cells, it was superior to other kidney injury markers for not being affected by age, sex, body weight, and inflammation. It is reported that CYSC used to detect AKI earlier than Crea [40]. In recent years, CYSC has been proven to be a potential new early biomarker and an independent predictor of mortality in AKI. In this study, the CV_I_ and CV_G_ of s-CYSC were 4.70% (95% CI, 4.23–5.29) and 14.07% (95% CI, 11.25–18.81), respectively, and the results were similar to those published on the website [11, 38]. The CV_I_ and CV_G_ of u-CYSC were 59.85% (95% CI, 53.86-67.34) and 57.80% (95% CI, 46.19-77.26), respectively. CYSC can be freely filtered by the glomerulus and is completely reabsorbed and decomposed in the proximal kidney tubules; however, it cannot be secreted into the lumen by the kidney tubules. Therefore, a large amount of CYSC does not appear in urine. The urine CYSC content in our study was very small or not, and we estimated BV by very low urinary CYSC levels.

NGAL has been recently used as a biomarker for the early diagnosis of AKI. It has the advantages of early, rapid, highly specific, and sensitive detection, which are less affected by other factors, and has attracted some attention in the field [7, 41]. NGAL is considered a "troponin of the kidney" [42, 43] because of its excellent performance for the early detection of AKI after surgery. In this study, the CV_I_ and CV_G_ of s-NGAL and u-NGAL were 11.64% (95% CI, 10.44–13.16) and 22.20% (95% CI, 17.74–29.68) and 61.82% (95% CI, 55.22–70.22) and 24.46% (95% CI, 19.54–32.69), respectively. The EFLM has not published a report on the BV of NGAL and this study fills this gap.

RCV is a statistical concept introduced in clinical judgment of two consecutive test results. When evaluating the change of test results, the numerical change of the results should be the sum of the inherent changes of the two test results. When assessing the changes in the test results, when the difference between the consecutive test results exceeded the RCV, the change in the results remained clinically significant even though the results were still within the reference interval. If the two test results are less than the RCV value of the laboratory, even if beyond the reference value range, it is not necessary to determine the abnormal results immediately, it is recommended to review in a few days. In this study, the RCV of kidney injury markers s-Crea, s-β2MG, s-CYSC, s-NGAL, u-Crea, u-β2MG, s-CYSC and u-NGAL were 21.88%, 29.31%, 15.32%, 33.09%, 131.73%, 168.01%, 206.59%, 172.90%.

II =CV_I_ / CV_G_. When II> 1.4, it means that the individual specificity of the test item is low, the variation between individuals is less than the variation of individuals themselves, and any small change in the patient's physiological state immediately exceeds the reference value range, indicating that the reference value range can be used to evaluate the continuous change in individual results, which is suitable for population screening. When II<0.6, it means that the individual specificity of the test item is high, the variation between individuals is greater than the variation of individuals themselves, and the reference value of the reference interval is limited. As a result, the test results of patients with abnormal results often fall within the reference interval representing the variation between individuals. The results of this study showed that the serum marker II value of kidney injury were less than 0.6, and the urine marker II value was greater than 1.0.

This study had some limitations. First, the evaluation of the short-term biological variation in markers was performed using in young healthy individuals within 24 hours. The differences in biological variation with respect to markers between children and the elderly or between individuals of different nationalities, specific disease statuses, detection methodologies and regions were not considered. Second, this study did not stratified for age and was biased towards young adults. Third, this study did not adjusted for urinary Crea. Further studies are needed.

## Conclusions

This study followed a rigorous protocol from 31 healthy subjects with narrow BV, providing the basis for biological variants in kidney injury markers s-Crea, s-β2MG, s-CYSC, s-NGAL, u-Crea, u-β2MG, u-CYSC and u-NGAL within 24 hours in healthy populations. The data of our study showed lower serum CV_I_ than urine CV_I_, serum CV_G_ higher than CV_I_, and urine CV_G_ lower than CV_I_. Urine marker II for kidney injury is higher than serum marker and is suitable for evaluating clinical decision-making with a population-based reference interval, while serum is more reasonable to evaluate them clinically using an individual-based reference interval or RCV.

## Data Availability

The datasets used and analysed during the current study are available from the corresponding author on reasonable request.

## Abbreviations

AKI: Acute kidney injury
CKD: Chronic kidney disease
GFR: Glomerular filtration rate
s-Crea: Serum creatinine
s-β2MG: Serum β2-microglobulin
s-CYSC: Serum cystatin C
s-NGAL: Serum neutrophil gelatinase-associated lipocalin
u-Crea: Urine creatinine
u-β2MG: Urine β2-microglobulin
u-CYSC: Urine cystatin C
u-NGAL: Urine neutrophil gelatinase-associated lipocalin
CVA: Analytical variation
CVI: Within-subject biological variation
CVG: Between-subject biological variation
II: Individual index
RCV: Reference change value
KDIGO: Kidney Disease Improving Global Outcomes
EFLM: European Federation of Clinical Chemistry and Laboratory Medicine
